# Future heat stress to reduce people’s purchasing power

**DOI:** 10.1371/journal.pone.0251210

**Published:** 2021-06-10

**Authors:** Kilian Kuhla, Sven Norman Willner, Christian Otto, Leonie Wenz, Anders Levermann

**Affiliations:** 1 Potsdam Institute for Climate Impact Research, Potsdam, Germany; 2 Institute of Physics, Potsdam University, Potsdam, Germany; 3 Mercator Research Institute on Global Commons and Climate Change, Berlin, Germany; 4 Department of Agricultural and Resource Economics, University of California, Berkeley, California, United States of America; 5 Columbia University, New York, New York, United States of America; Istanbul Medeniyet University: Istanbul Medeniyet Universitesi, TURKEY

## Abstract

With increasing carbon emissions rising temperatures are likely to impact our economies and societies profoundly. In particular, it has been shown that heat stress can strongly reduce labor productivity. The resulting economic perturbations can propagate along the global supply network. Here we show, using numerical simulations, that output losses due to heat stress alone are expected to increase by about 24% within the next 20 years, if no additional adaptation measures are taken. The subsequent market response with rising prices and supply shortages strongly reduces the consumers’ purchasing power in almost all countries including the US and Europe with particularly strong effects in India, Brazil, and Indonesia. As a consequence, the producing sectors in many regions temporarily benefit from higher selling prices while decreasing their production in quantity, whereas other countries suffer losses within their entire national economy. Our results stress that, even though climate shocks may stimulate economic activity in some regions and some sectors, their unpredictability exerts increasing pressure on people’s livelihood.

## Introduction

Anthropogenic greenhouse gas emissions have already led to an increase in global mean temperature by about 1°C compared to pre-industrial levels [1–3]. Due to inertia in the climate system, the planet will continue to warm over the next two decades even under possible emission reductions [4]. With global mean temperature rising, the number and intensity of extreme heat events are expected to increase [5, 6]. A growing body of empirical literature suggests that rising temperatures have various socio-economic impacts by affecting, for example, agricultural output [7], human health [8], energy supply [9, 10], income [11, 12], and labor productivity [13, 14]. Nevertheless, on a global scale there is not yet satisfying coverage on the overall production loss estimates due to heat stress [15].

Labor productivity, which is strongly impacted by heat stress [16], has been shown to decrease quasi-linearly with daily mean temperatures exceeding 27°C [17, 18]. Possible relations to physiological heat stress make this particularly relevant for outdoor sectors such as forestry, mining, and construction, where workers are strongly exposed to ambient temperature [19–21]. Stronger efforts to mitigate climate change are needed to avoid further increase in heat stress [22–24], but warming is unlikely to be halted in the next two decades. Without specific adaptation measures—which come at their own costs [25]—an increasing number of hot days due to globally rising temperatures will thus lead to temporarily reduced productivity of these sectors.

These losses can be substantial; for instance, the Russian heat wave in 2010 caused output losses of about USD 15bn in the Russian economy alone [26]. The business interruptions associated with such a strong shock to a local economy (“direct losses”) can additionally spread to other sectors and regions via demand and supply cascades as well as associated price signals [27–30]. Resulting indirect losses [31, 32] can be particularly high in today’s densely connected global economic network. However, there can also be gains through market adjustments, which effectively redistribute production in the short run [33–35]. Thus, depending on the structure of the economic network [36], the flexibility of the economic actors within that network [37], and the strength and regional pattern of the heat stress signal, direct losses can be both, amplified and mitigated through economic response dynamics.

In this study, we estimate the short-term effects of heat stress-induced reduction in labor productivity and corresponding output losses for 2020–2039 in comparison to 2000–2019. The effects are short-term in the sense that they are on the scale of days to weeks arising from direct productivity reduction beyond a daily temperature threshold on each individual day. In particular, we show the short-term repercussions of these specific shocks in the global economic network in terms of indirect production losses, price changes, and effects on consumption on a national and global scale. We show that the latter can be substantial and are likely to increase in many regions. We add up these daily economic repercussions to annual values and depict a long-term trend of these aggregated short-term effects for the next double decade. For that, we employ a loss-propagation model including a total of 7, 236 economic agents which form a network of about 1.8 million interconnections. Results are thus under the economic structure of 2012 in the absence of adaptation.

## Materials and methods

### Temperature data

We use time series of daily mean temperature for the period 2000–2039 as a physical driver for economic outages. The projected temperature data are provided by four global climate models (GCMs) of the CMIP5 [38] ensemble (HadGEM2-ES [39], IPSL-CM5A-LR [40], MIROC5 [41], GFDL-ESM2M [42]), which have been bias-corrected within ISIMIP [43] (project phase 2b) towards an observation-based data set using a trend-preserving method [44] at a spatial resolution of 0.5° × 0.5°. Using a bias-correction, extreme weather phenomena, which in the GCMs tend to be averaged out, can be better represented in the time series; thus, the daytime temperatures do not correspond to the historical values, but are well represented in their statistics over the ensemble (for consistency we also use ISIMIP2b output (historical scenario) for the historic period). For each model we use the representative concentration pathways (RCPs) 2.6 and 6.0. This combination results in an ensemble of eight daily temperature time series and thus eight direct output loss time series. Although the emission path for the next two decades is largely determined due to the inertia of the climate system, the usage of different RCPs provides a larger simulation ensemble.

### Population data for distribution of production

Population data rely on the Population Count v4.11 for the year 2015 of the GPW data set [45] aggregated to the resolution of the physical input data (0.5° × 0.5°). We use these population data as a proxy for the distribution of production of a region over a gridded area, meaning that a cell where *x*% of a region’s population lives accounts for *x*% of its production as well. Thus, a heat wave over a densely populated area causes a higher direct loss of production than over sparsely populated area. As a region mask we use GADM data [46], which we rasterize to the same resolution (0.5° × 0.5°) advanced in coastal areas to account for inaccuracies at shape boundaries.

### Direct output losses due to heat stress

To obtain a region and sector specific damage function, we employ the empirical relationship between daily temperature and production capacity derived by Hsiang et al [17]. The production reduction bases solely on short-term performance shortfalls and not on longer-term structural damage, such as destroyed infrastructure. Though originally focuses on Caribbean countries, we transfer the productivity-temperature-correlation to all countries in the world. In our study, we take a look at the spatio-temporal responses of the global economic network. For this, a linear temperature-productivity-relation approach for every region makes slightly less assumptions than a non-linear relation. We translate the local daily mean temperature to direct output production losses within a regional sector. Every grid cell *r* where the daily mean temperature *T*_*r*_(*t*) at day *t* surpasses 27°C suffers a linear reduction *α*_*s*_ in its productivity *p*_*s*,*r*_(*t*) per °C beyond 27°C for the sectors {*s*}:

- agriculture (−0.8 p.p./°C),
- fishing (−0.8 p.p./°C),
- mining and quarrying (−4.2 p.p./°C),
- hotels and restaurants (−6.1 p.p./°C),
- wholesale trade (−6.1 p.p./°C),
- and others (−2.2 p.p./°C)

(the unit p.p./°C corresponds to percent points per additional degree Celsius).

Thereby we only consider sectors for which statistically significant results in reduction of labor productivity have been found [17] as well as the agriculture and fishery as important sectors (impact on these is comparatively small, though). Thus, we exclude the sectors transport, communications, construction and manufacturing for which the empirical results were not statically significant.

Thus, we have a perturbed productivity of

$$\begin{array}{c} p_{s,r}\left(t\right)=1-\alpha_{s}\left(T_{r}\left(t\right)-27\;{}^{\circ}C\right)\;\text{for}\;T_{r}\left(t\right)\geq 27\;{}^{\circ}C. \end{array}$$ (1)

The perturbed productivity per sector *s* of each cell *r*, which belongs to a region *R*, is aggregated to the daily perturbed productivity per sector *s* of a region *R* weighted by the population distribution:

$$\begin{array}{c} p_{s,R}\left(t\right)=\frac{\sum_{r\in R}p_{s,r}\left(t\right)P_{r}}{\sum_{r\in R}P_{r}}, \end{array}$$

with *P*_*r*_ being the population of cell *r*.

Absolute output losses are then determined by multiplying the perturbed productivity with the baseline production of that region (see next section).

### Economic network

As the underlying baseline for the global economic network, we use multi-regional input-output (MRIO) tables of the EORA simplified data set v199.82 [47]. The build-up and harmonization of the input-output table base on certain assumptions, which barely affect the general structure of the flows, but rather their details. In our sensitivity analysis we show that our results are stable with respect to changes of individual flows. The commodity flows in the MRIO table are provided as monetary flows (in USD of the data year) between regional sectors. The economic baseline network is build from the EORA MRIO table for the year of 2012. Flows smaller than 1000$/year and regional sectors with negative value added are removed from the network. A few regions (Belarus, Guyana, Moldova, Zimbabwe) with inaccurate data basis, causing partially an extremely unrealistic temporal evolution, were neglected in our analysis. Because of their large economic power, we further disaggregate [48] the United States of America and China into 51 states and 32 provinces using gross regional product (GRP) data [49, 50] of the individual states and provinces, respectively. We thereby split the flows of goods and services from and to a subregion according to the share of the subregion’s GRP of the total country’s GDP. Each economic sector in a nation, US state or Chinese province, which we refer to as regions for simplicity, forms an individual agent or “regional sector” in the loss-propagation model Acclimate (see below). In addition to mapping the flows of goods and services, the EORA MRIO table is used to calculate the baseline production of each regional sector. The resulting economic network comprises 27 sectors (including one consumer sector) and 268 regions, a total of 7, 236 agents.

### Indirect production losses—The Acclimate model

We use the agent-based loss-propagation model Acclimate [51] to project the dynamics of the global economic network and derive overall production and consumption losses. This anomaly model revolves around the economic baseline given by the multi-regional input-output data. The direct output losses, given as short-term production reduction, impact this economic baseline network; Acclimate then simulates the behavior of firms (regional sectors) and consumers when perturbed from the baseline by a demand, supply, or price shock. In that, each regional sector, represented by a node in the input–output network, individually maximizes its profit by choosing the optimal production level and corresponding upstream demand as well as the optimal distribution of this demand among its suppliers.

In more detail, every time step consists of three subsequent decision points for each agent. In the first sub-step, a firm calculates its current production level in order to maximize its profit (difference between revenue and costs) under the current supply and demand circumstances. Any restrictions, such as limited production capacity or limited number of input goods, are taken into account. In particular, production costs increase non-linearly if firms extend their production beyond baseline quantities. In their sales prices firms are bound by the demanded requests and price offers received from their purchasers. Ordering these orders by descending price results in a concave revenue curve. Similar to firms, consumers decide on their consumption, but following the perceived prices according to their respective consumption elasticity. In the second sub-step agents update their expectations for the next time step according to goods received in the first sub-step. In the last sub-step, each agents decides via cost-minimization the distribution of demand among its suppliers in terms of quantity and price. This is based on supplier’s offer prices as well as expectations on their cost curves. Thus prices are endogenous non-equilibrium prices that are can differ locally for each supplier-purchaser pair. Supplied goods are transported between agents following a distance-based delay. Together with storage inventories, these transport pathways act as buffers for supply shocks.

Overall, the model dynamics focuses on the perturbations around a baseline equilibrium. Whereas this baseline is assumed to be optimal on longer time scales the endogenous local price changes as well as supply and demand mismatches are resolved explicitly over time. In the disaster aftermath, these relax back to the unperturbed baseline equilibrium over a timescale determined by the market. Computed losses thus account for price effects such as demand surge and supply shortages. With its focus on short-term perturbation dynamics the model does not include structural changes in the global trade network, such as investments, relocation of production or the establishment of new trade relations. A comprehensive model description of Acclimate is provided in Otto et al [51].

### Consumer price elasticity

The consumers’ reaction to price changes depends on consumed commodity and services as well on their own economic background. To reflect this in our model, the consumer price elasticity differs among regions and sectors. For that, we divide the regions into four groups, income levels, corresponding to their gross national income (GNI) per capita (GNI/pc) (S2 Table) (low, lower-middle, upper-middle, high income level). As income rises, the flexibility to react to prices increases which enables countries with a higher GNI/pc to respond more resiliently to price changes. The World Bank provides an annually and inflation-adjusted definition of income levels and country-specific data [52], which we use as a basis for our classification for the year 2012. We do not consider other national socio-economic structures (education, wealth distribution, etc.) in this study. Accordingly, we base the classification on average income only.

Within the Global Trade Analysis Project (GTAP) [53], target income elasticities of demand have been calculated for 140 regions and 10 commodity classes [54]. The regions of EORA and GTAP do not match perfectly. For non-GTAP-countries we assign the consumption price elasticities via the respective income level group specific parameter. To link the different sectoral resolutions, we map our economic sectors to the GTAP classes and then group them into three categories: vital, relevant, and other. The categorization of the sectors used is given in S1 Table. The more life essential goods or services are, the less flexible consumers can be in responding to price fluctuations. Using the three sector categories and the World Bank’s country classification, we assign a specific consumer price elasticity from the GTAP data set [55] for any pair of level income and sector category (S3 Table). We do not consider cross-sector elasticity as one can assume low to no substitutability for the large sector classes we use (S1 Table).

### Limitations

Into this short-term loss-propagation model labor productivity shocks enter as direct reductions in productivity, but do not take into account longer-term damage, such as destroyed infrastructure. As we focus on these short-term shocks and the corresponding economic repercussions, we assume that there is no investment and no explicit capital on these time scales. Also neither firms nor consumers have the explicit notion of savings. However, on a daily time scale, the effects of corporate growth or relocation of production are small compared to external production constraints [35]. With its agent-based short-term dynamics, the Acclimate model is particularly suited to assess the global distribution of consequences of unanticipated short-term shocks such as those caused by heat stress. Nevertheless, this study only focuses on a single impact channel of climate change disturbing the global economy. Naturally, this occurs additional to other economic activity. Our results should thus not be interpreted as quantitative projections, but show trends between the last and upcoming double decades.

## Results

We find (i) increases in direct output losses, (ii) a very heterogeneous distribution of overall total production losses and gains when incorporating price changes and other indirect effects (with gains in a majority of countries), and (iii) a differently distributed consumption response with almost all regions reducing consumption while increasing expenditure. In the following, we give detailed results for the median of the ensemble of four climate models and two representative concentration pathways each. These results should not be interpreted as literal projections, but show trend and magnitude of the higher-order effects of this one particular impact channel of climate change—under the current economic structure and in absence of adaptation.

### Direct output losses

**Fig 1:**
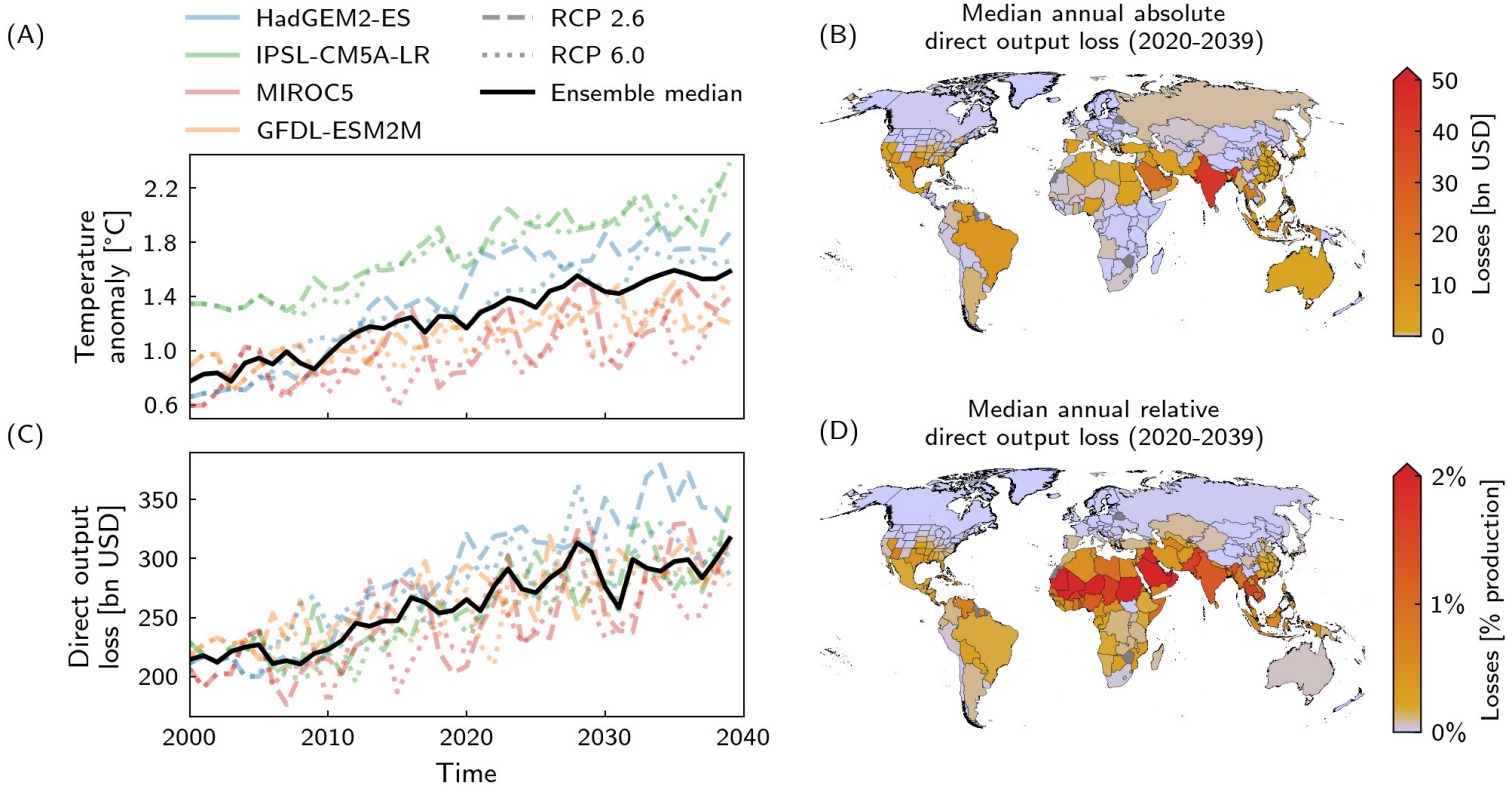
Global mean temperature anomaly and heat stress-induced direct output losses. A,C: Temporal evolution of A global mean temperature anomaly relating to pre-industrial level and C heat stress-induced direct output losses for the four climate models, HadGEM2-ES (blue), IPSL-CM5A-LR (green), MIROC5 (red), GFDL-ESM2M (orange) and RCP2.6 (dashed) and RCP6.0 (dotted), and ensemble median (black line). The higher temperature anomaly of climate model IPSL-CM5A-LR compared to the other climate models is due to the relatively lower model-internal pre-industrial temperature. B,D: Regional maps of B absolute and D relative annual direct output loss due to heat stress based on the respective regional projected median for 2020–2039. Regions with an absolute or relative direct annual output loss below USD 1bn or 0.2% of baseline (unperturbed) production are depicted in light purple.

According to global climate models, global mean temperature increases by about 0.8°C between 2000 and 2039 and as a consequence direct output losses increase by 47%—if no further adaptation measures are taken (Fig 1A and 1C). Within the next two decades, the global direct output loss will increase by 24% (in 2039 compared to 2020). This corresponds to an increase in global direct output losses of about USD 127bn per degree of global warming. These local heat stress-induced losses are heterogeneously distributed across regions (Fig 1b). For all maps shown in this paper, shapes of countries are based on GADM data [46]. About 84% of all regions exhibit an annual median output loss of less than USD 1bn p.a. in 2020–2039 whereas several major economies experience substantially higher output losses. Among those are Saudi Arabia (USD 22bn p.a.), China (USD 29bn p.a.), USA (USD 40bn p.a.), and India (USD 44bn p.a.). In terms of relative losses, i.e. a percentage of a country’s overall production level, countries of the Sahel, the Arabian Peninsula, and South Asia are impacted the strongest (Fig 1d). The global rise in temperature leads regionally to an increase in direct losses in the billions USD (e.g. in India, Saudi Arabia, or Mexico) or nearly double the direct output losses (e.g. in Northern America or Europe) within the next decades (S1 and S2 Figs).

#### Total production losses and gains

**Fig 2:**
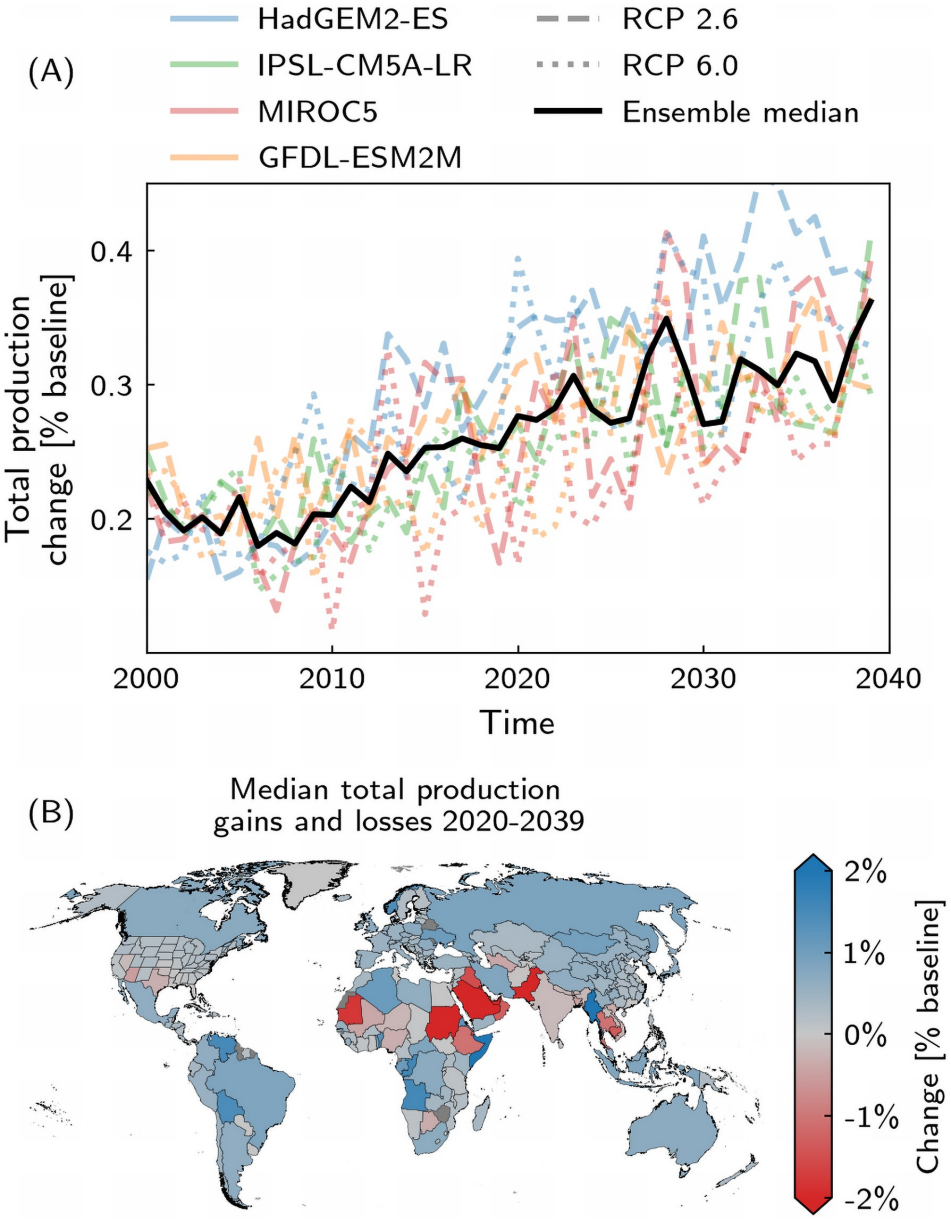
Total production losses and gains. A: Temporal evolution of global total production change for the four climate models, HadGEM2-ES (blue), IPSL-CM5A-LR (green), MIROC5 (red), GFDL-ESM2M (orange), and RCP2.6 (dashed) and RCP6.0 (dotted), and ensemble median (black line). B: Regional map of total production gains and losses based on the respective regional projected median for 2020–2039 and the full GCM–RCP ensemble. Quantities are given relative to the baseline (unperturbed) production.

**Fig 3:**
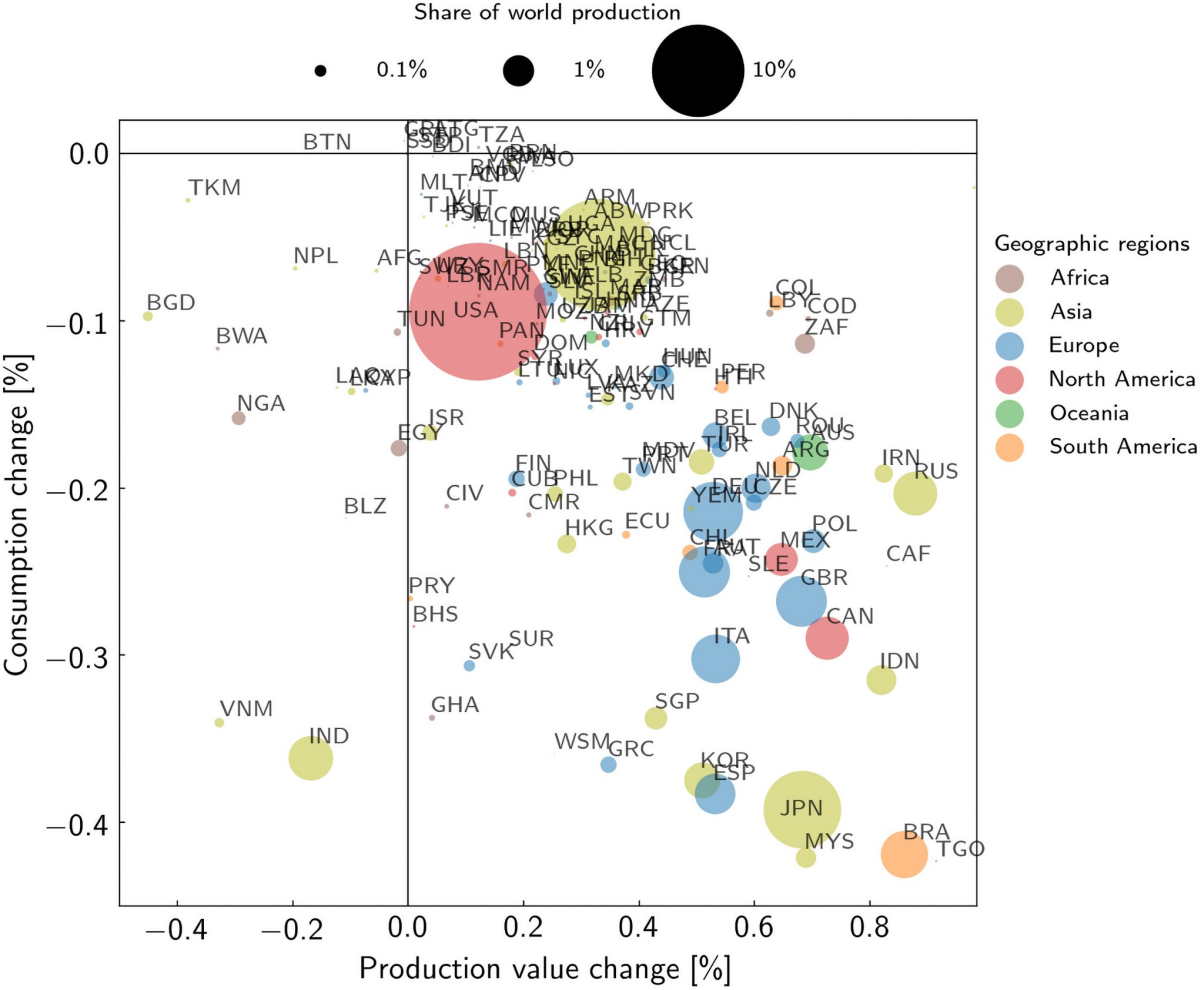
Consumption and total production change per country for the time period 2020–2039. The area of each dot is proportional to the corresponding country’s baseline (unperturbed) production. The dot colours denote the geographic regions (see S2 Table). Quantities are given relative to the baseline (unperturbed) production and consumption, respectively.

These direct output losses evoke a market response locally as well as across country borders. The heat stress-induced scarcity inflates prices of intermediate goods and services as well as consumer prices. On a global level, the increase in value of produced goods rises from 0.23% p.a. in 2000 to 0.36% p.a. in 2039 (Fig 2a). Again, there are large differences between countries. Some sectors in some countries even profit from heat stress-induced outages of their competitors when scarcity inflates the prices for their product and/or they receive more demand requests. For example, the US state of Texas and the country of Iraq have to cut back their production in the petroleum and non-metallic mineral sector, while Iran and Russia are ramping up their production in this sector. Many countries, including most G20 countries, are able to increase the value of their goods and services due to price effects despite direct output losses (Fig 2b). Economic relations are a relevant factor here. Although Spain and Greece suffer significant direct output losses (Fig 1), they are still increasing their production value through the European Single Market (Fig 2b). This is in contrast to Arizona and Texas failing to convert their direct output losses within the US economic system into total production gains. A few countries (e.g. Saudi Arabia, India, Thailand), suffer from many hot days in a year and therefore cannot benefit from a positive change in total production as well (Fig 3).

### Consumption losses and change in expenditure

Facing higher prices, consumers have a certain willingness and ability to increase their expenditure in order to reduce consumption losses. This behavior determines the increase in household expenditures alongside a price-driven reduction in material consumption. In our model, consumers’ willingness and ability to adjust their expenditures in response to rising prices is described by the consumption price elasticity. Since economic well-being and necessity of goods and services have a crucial role on consumption decision, we use a country and sector-specific consumption price elasticity (see Methods for a detailed description).

As a consequence, as global output decreases and prices inflate, consumption is diminished. However, our results show that this occurs regardless of the question if the country itself actually gains from increased prices in production value or not (Fig 3)—only a few economically small states show (very small) consumption gains. This is due to the strong global interconnectedness transferring heat-stress losses in terms of consumption also to countries not affected themselves. Though some of these countries can benefit in their production values, the consumption reduction can only partiality be compensated for; in most countries there is a higher share of production gain than consumption loss (Fig 3). Although in the three largest economic regions, USA, China, and the European Union, the heat stress-induced direct production losses are distributed heterogeneously within their regions (Fig 1B and 1D), the EU exhibits an enhanced consumption loss and total production gain (Fig 3). This may be due to the strong economic connection among US states and among provinces of China. These connections make it easier to compensate for production losses within these countries. Compared to the historical period (2000–2019), gains and losses of total production and consumption increase moderately in the future, but the qualitative pattern of the countries remains similar (Fig 3 and S3 Fig).

**Fig 4:**
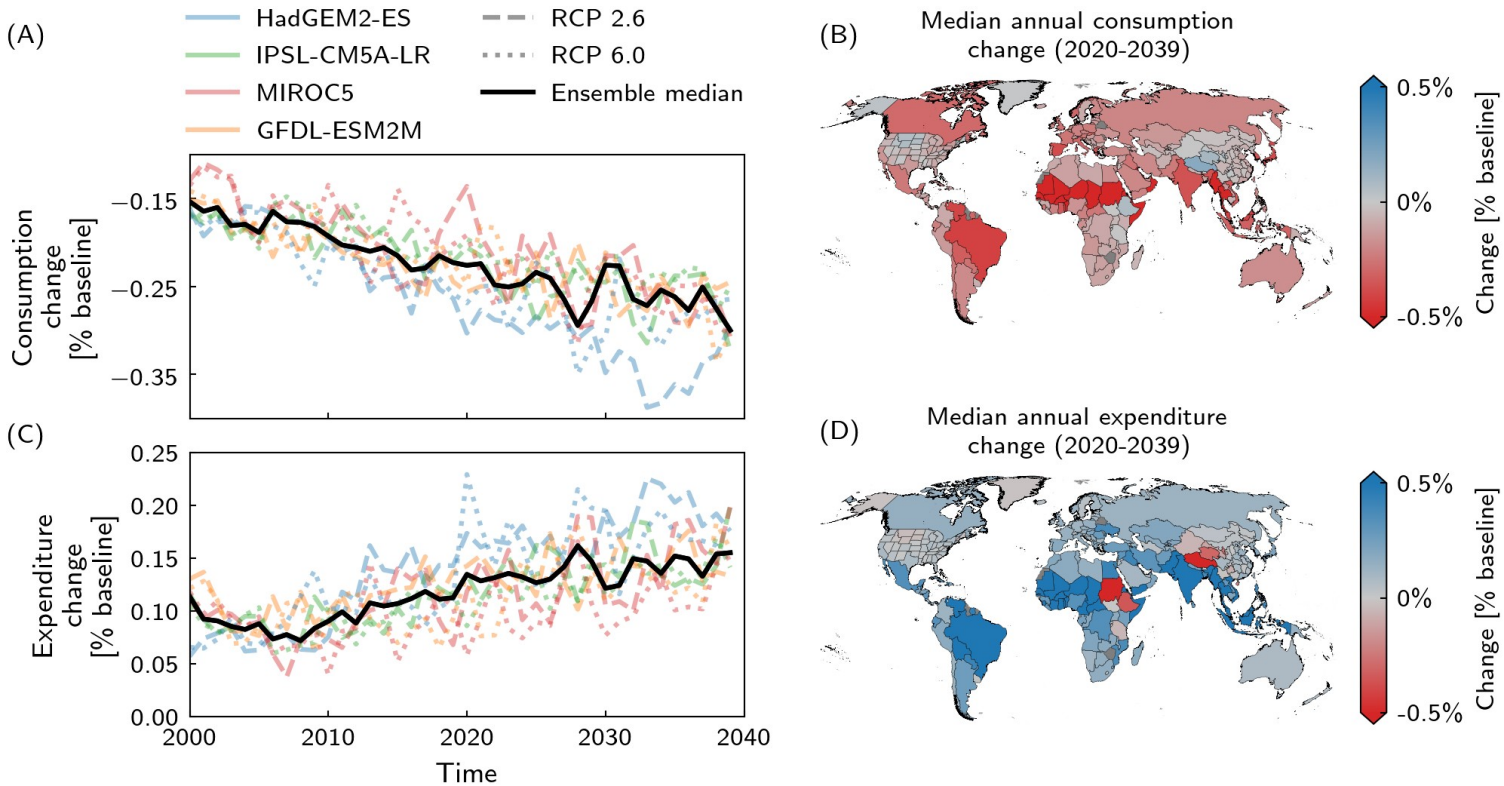
Consumption and expenditure change. A,C: Temporal evolution of A global consumption change and C global expenditure change for the four climate models, HadGEM2-ES (blue), IPSL-CM5A-LR (green), MIROC5 (red), GFDL-ESM2M (orange), and RCPs 2.6 (dashed) and 6.0 (dotted), and ensemble median (black line). B,D: Regional maps of B annual consumption change and D annual expenditure change based on the respective regional projected median for 2020–2039 and the full GCM–RCP ensemble. Quantities are given relative to the baseline (unperturbed) consumption and expenditure, respectively.

Global consumption decreases, i.e. heat stress-induced consumption losses increase from 0.15% p.a. in 2020 to 0.30% p.a. in 2039 (Fig 4A). These quantitative losses come at a higher consumer expenditure, which increases from 0.11% p.a. in 2020 to 0.16% p.a. in 2039 (Fig 4C). Per degree of warming, expenditure thus rises by 0.06 percentage points as consumption falls by 0.18 percentage points. These results are regionally fairly homogeneous (Fig 4B); about 93% of the global population lives in a region where consumer expenditures increase (Fig 4D). The pattern of pressure on consumers does not change between historical and future periods (S4B and S4C Fig).

Neglecting other economic benefits such as potentially higher wages because of increased production value (as most, though not all, countries with diminished consumption show), this comes at a decrease of household welfare. Overall, one can say that the heat stress shocks on productivity lead to a reduced overall economic efficiency which cannot be fully mitigated by market flexibility.

### Changing the underlying economic network

**Fig 5:**
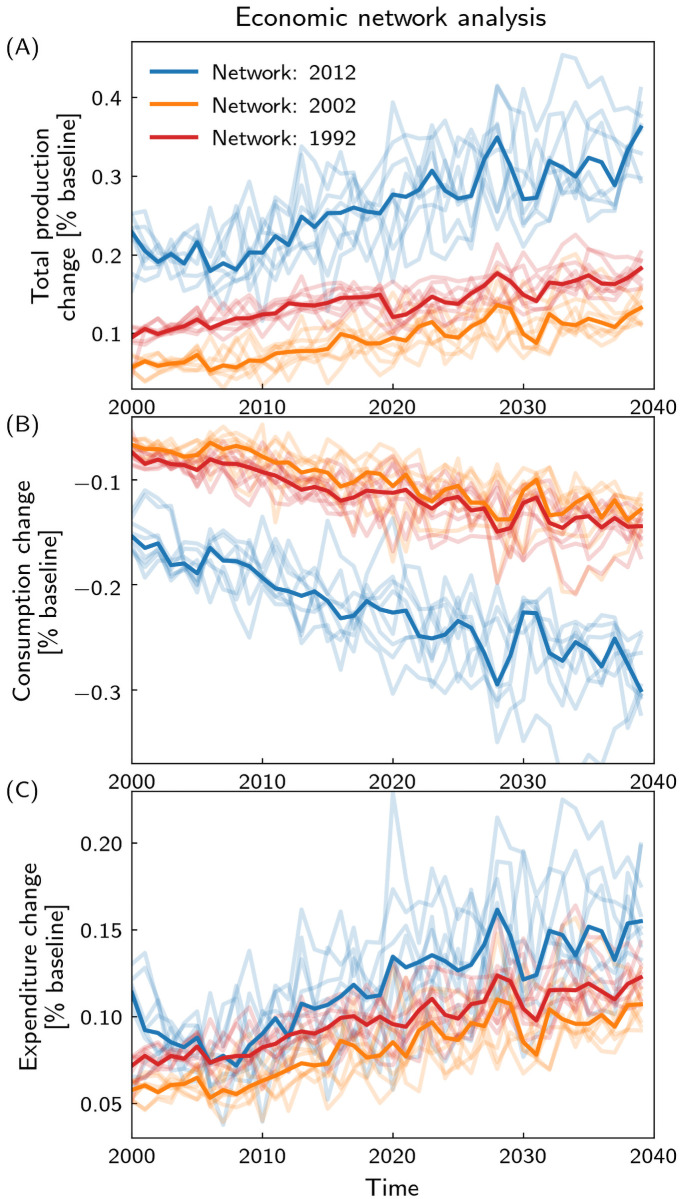
Total production, consumption, and expenditure change for economic networks of 2012, 2002, and 1992. Temporal evolution of A total production changes, B consumption change and C expenditure change for economic networks of 2012 (blue), 2002 (orange) and 1992 (red). Ensemble members and median are depicted in light and thick lines, respectively.

The results above all assume a constant baseline structure of the global economic network, i.e. they are to be interpreted “given the world economy of 2012”. Nevertheless, our results are qualitatively robust to the network used when compared to results with the network structures of 1992 and 2002 (Fig 5). Quantitatively, however, earlier network structures show a smaller response, in particular in the total production changes, due to their much smaller interconnectedness [36]. Interestingly, consumer expenses are similar for all three networks, though consumption losses are by far highest for the 2012 network (Fig 5B and 5C). For the future world economy assuming further globalization of supply and trade chains, one would thus expect an even stronger response to heat stress even without climate change.

### Results robust against parameter choice

**Fig 6:**
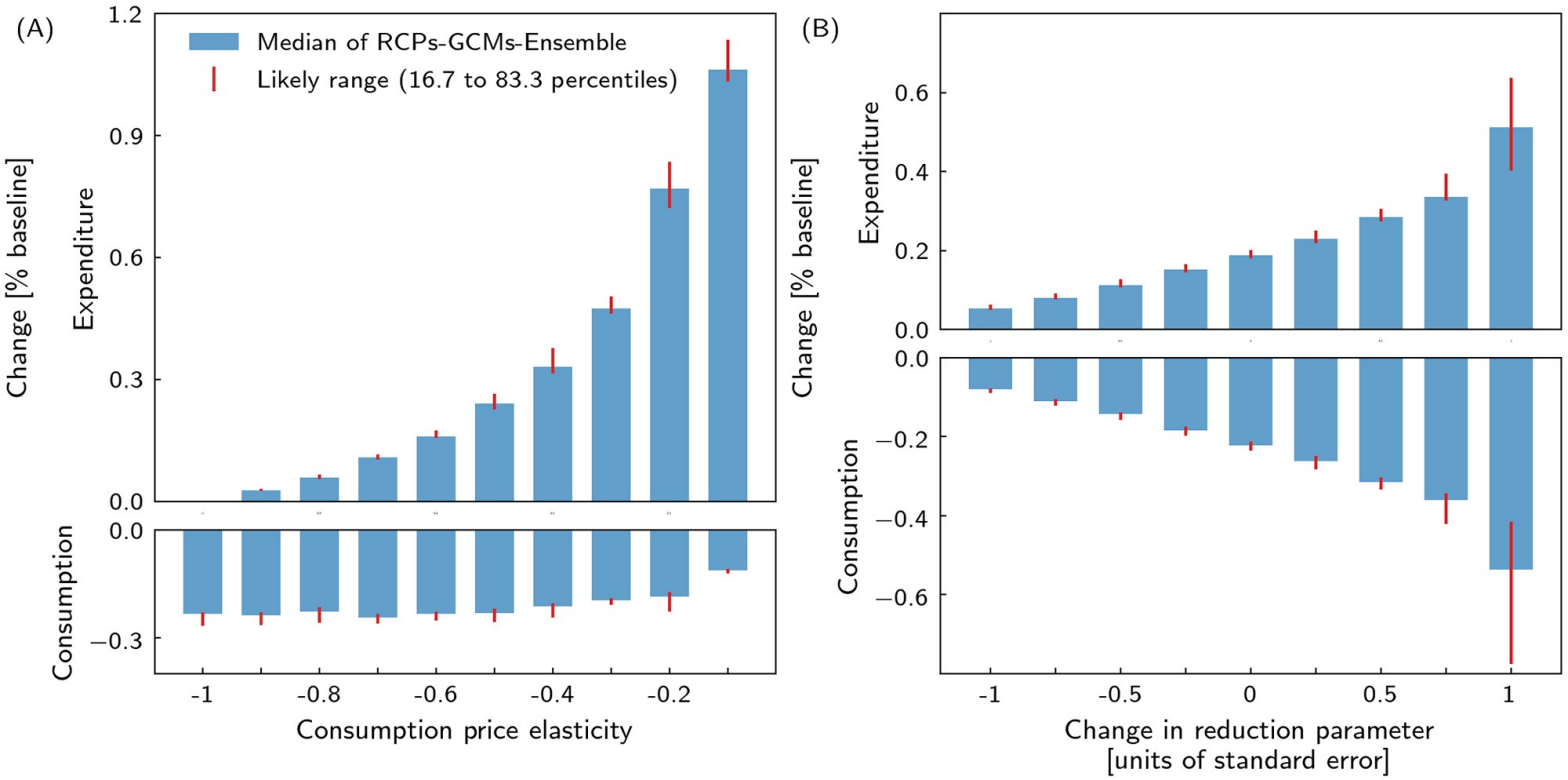
Results are robust against choice of consumption price elasticity and temperature-productivity-uncertainty. Median of RCPs-GCMs-Ensemble and likely range (16.7 to 83.3 percentiles) of heat stress-induced changes in consumer expenditure and consumption in 2039 relative to unperturbed baseline for A different consumption price elasticities and B change in intensity of productivity reduction due to heat stress.

Central to the overall consumer behavior in our model is the consumption price elasticity. To assess its influence on our results, we conduct a sensitivity analysis sampling this parameter for every consumer in a range from −1.0 (highly flexible to price changes) to −0.1 (almost disregarding price changes) (Fig 6A). In the analysis, all consumers have the same consumption parameter so that results are directly comparable. In our parameter study, we use the eight direct output loss time series to shock Acclimate and we focus on the global consumption losses and expenditure raise in 2039. On the one hand, global expenditure rises with increasing inflexibility. This is reasonable to expect as consumers are less likely to accept price increases with greater flexibility. On the other hand, global consumption losses due to heat stress hardly change. So the future heat stress-related pressure on people’s purchasing power is largely insensitive to the choice of the consumption price elasticity.

This study uses quasi-linear direct production reduction based on empirical econometric values [17]. A sensitivity analysis of this functions shows that the trend of our results persists against changes in the direct production reduction (Fig 6B). For that, the damage function parameters are sampled in their standard error range [17]. Global consumption losses and expenditure rise increase with higher direct output reduction. An increased sensitivity to heat stress leads to more disturbed production. The higher price increase due to greater scarcity is passed on to the consumer, who spends more and consumes less. Overall, our qualitative results are robust under a wide range of values of those parameters.

## Discussion

Our results are in line with studies looking at the direct effect of heat stress [17, 18] and its consequences on the local economy [56]. We hereby give an additional global perspective including the impact of heat stress on short-term price changes as well as supply and trade chains. Besides a chance for “building back better” after an economic shock as suggested by other studies [57], we also identify a potential positive side effect for many less affected regions when considering shifting of demand and supply. We show that this effect, however, comes at the expense of the consumer. On a longer time scale, e.g. by affecting economic growth, also production might be affected negatively [58]. A stimulating effect of productivity shocks would also cease for stronger disasters, such as floods, which have an overall negative effect on directly as well as indirectly affected regions [37]. It is important to note that our study focuses on consecutive extreme events [59] rather than singular shocks; locally, heat stress constitutes many small but lasting background shocks to the economy, partly even simultaneously at different regions, and thus puts a persistent pressure on the global supply network. Building on our results, further studies could provide estimates on sub-national distribution of heat induced consumption losses as well as on economic impacts due to heat stress for the mid- and end-century.

Since the interaction between consumption, production, and price is based on a complex chain of measurable factors (e.g. income) and psychological factors (e.g. expectations) [60], any modeling of these variables is necessarily based on generalizing assumptions. Here, we specify the main assumptions of our study and and discuss their adequacy. Regional daily temperatures from climate projections and resulting possible heat-related losses are not exact forecasts but rather serve as exemplary conditions here. For that, we use an ensemble of individual loss time series derived from eight different RCPs–GCMs combination. Since the trend and magnitude of the results are consistent within the ensemble, we observe that our results are robust with respect to the uncertainties of climate projections and independent of individual daily weather conditions. In this study we focus specifically on the impact on the economy and consumers due to short-term heat stress-related production losses. Therefore we opt a daily temperature-production-correlation. In particular, we generalize a heat impact shown only for Caribbean countries [17] to every region of the world. This assumption is supported by research showing that climatic conditions have non-linear effects on human productivity in any country [18, 21]. Similar to Hsiang et al [17], other studies are limited to a specific region as well [61]. With this damage function we show the trend evolution of consumption losses in the past and future double decade.

Apart from physical and econometric uncertainties, there are also constraints on economic and human behavior modeling due to their complexity. In order to properly interpret our results in the light of the limits of our study, we clarify the main boundaries of our modeling in the following. For one, we are simulating short-term economic repercussions, so we do not need to consider longer-term socio-economic changes, such as investment strategies of firms or new trade linkages. In particular, we use the economic trade network of 2012 as the fixed socio-economic baseline. Our sensitivity analysis on the networks depict that the trend of our results is robust against different economic networks. Additionally, the results of our analysis imply that in a more interconnected world trade the impact of heat stress is likely to increase. Furthermore, we explicitly neglect adaptation, since we do not want to make assumptions on possible adaptation measures and their effects. Thus we can interpret our results as a more direct response of the global supply network. In this respect, our results indicate that adaptation measures to heat stress in production are necessary. Global warming makes such measures even more vital.

## Conclusion

Our results must not be interpreted as literal and comprehensive predictions of the effects of future heat stress events. They may rather serve as a qualitative prediction with quantitative predictive skill limited to the order of magnitude of the signal. Overall, they highlight that heat stress will, without adaptation measures, increase direct output production losses, regionally and globally. Those unexpected climatic events such as heat waves can reduce the welfare of consumers, even if economic key indicators such as production value, and thus nominal GDP, suggest beneficial economic effects in many regions. This indicates that intensified and more frequent heat waves constitute an increasing threat to prosperity of nations in the near future.

## Supporting information

S1 Fig Heat stress-induced median annual direct output losses for the historic time period 2000–2019. Regional maps of A absolute and B relative annual direct output loss due to heat stress based on the respective regional bias-corrected median for 2000–2019. Regions with an absolute or relative direct annual output loss below USD 1bn or 0.2% of baseline (unperturbed) production are depicted in light purple, respectively.
(TIF)

S2 Fig Projected increase of heat stress-induced annual direct output losses between historic (2000–2019) and future (2020–2039) period. A Absolute annual increase of regional direct output losses of period 2020–2039 compared to 2000–2019. B Increase of direct output losses in the future period in terms of losses in the historic period.
(TIF)

S3 Fig Consumption and total production change per country for the historic period 2000–2019. The area of each dot is proportional to the corresponding country’s baseline (unperturbed) production. The dot colors denote the geographic regions (see S2 Table). Quantities are given relative to the baseline (unperturbed) production and consumption, respectively.
(TIF)

S4 Fig Total production, consumption, and expenditure change for the historic period 2000–2019. Median annual change of A total production, B consumption and C expenditure relative to the unperturbed baseline.
(TIF)

S1 Table Sectors used in the simulations. For sectors prone to heat stress-induced productivity loss the respective reduction factor (see Methods) is given in the last column.
(PDF)

S2 Table Regions used in the simulations. Income level corresponds to Gross National Income per capita (GNIpc) of 2012.
(PDF)

S3 Table Consumption price elasticities per income level and sector category. Values are based on GTAP [55].
(PDF)
