# Supplementary figures and images for: Future heat stress to reduce people’s purchasing power
Source: PLoS One. 2021 Jun 10;16(6):e0251210. doi: 10.1371/journal.pone.0251210 (PMC8191966; doi:10.1371/journal.pone.0251210)

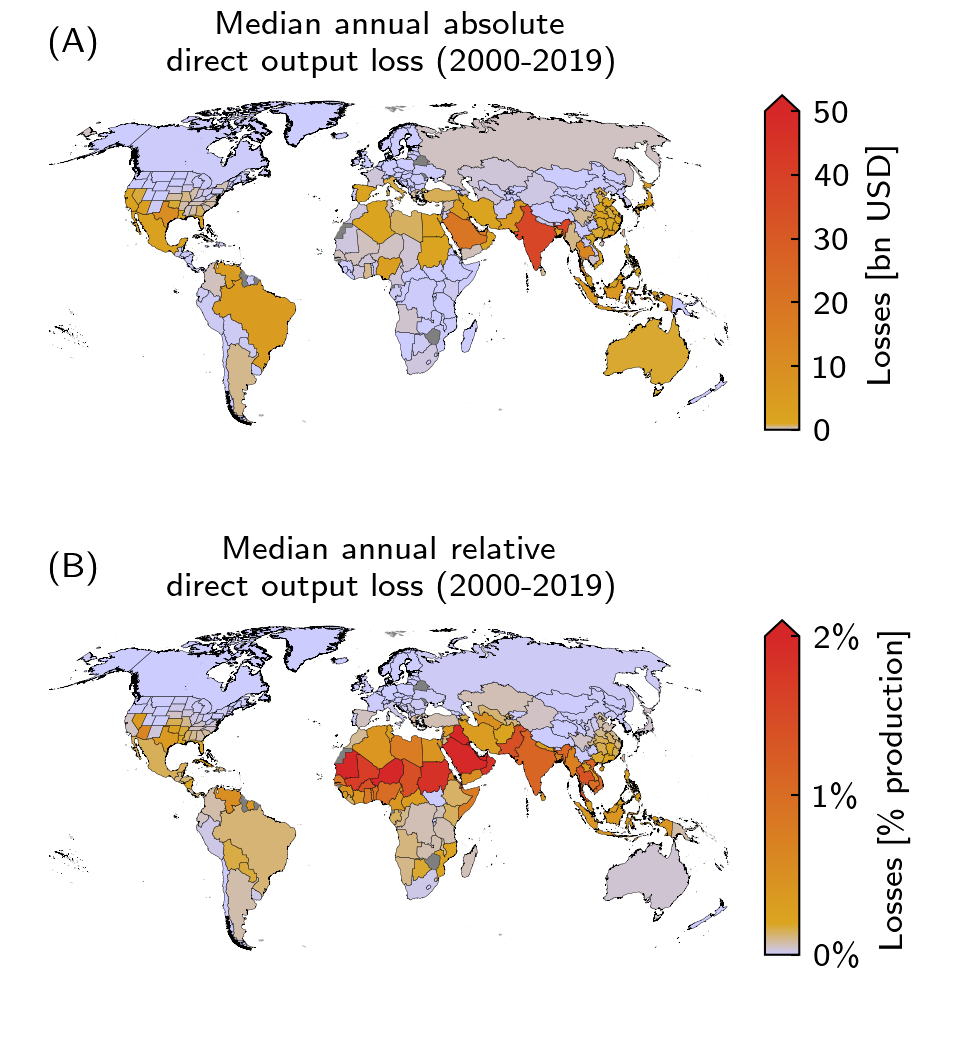

Supplement: S1 Fig — Regional maps of A absolute and B relative annual direct output loss due to heat stress based on the respective regional bias-corrected median for 2000–2019. Regions with an absolute or relative direct annual output loss below USD 1bn or 0.2% of baseline (unperturbed) production are depicted in light purple, respectively. (TIF) [file pone.0251210.s001.tif]

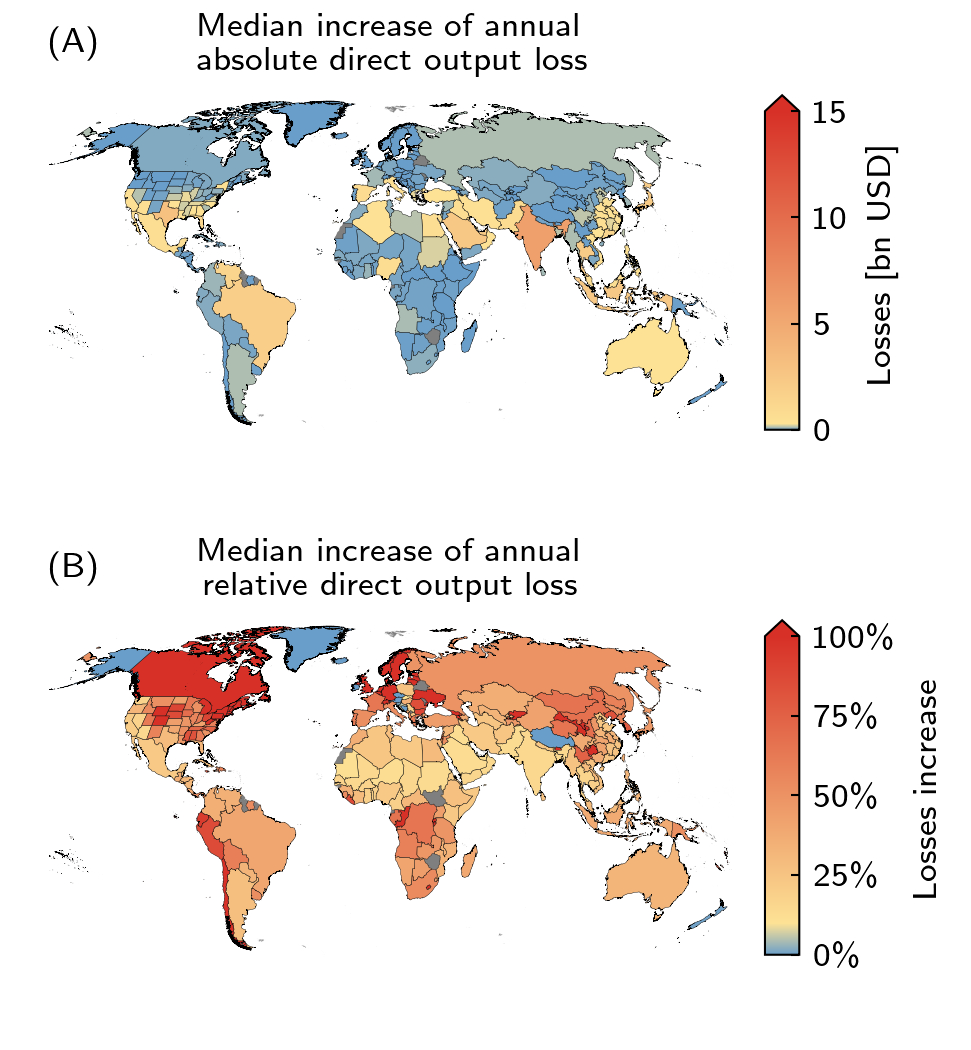

Supplement: S2 Fig — A Absolute annual increase of regional direct output losses of period 2020–2039 compared to 2000–2019. B Increase of direct output losses in the future period in terms of losses in the historic period. (TIF) [file pone.0251210.s002.tif]

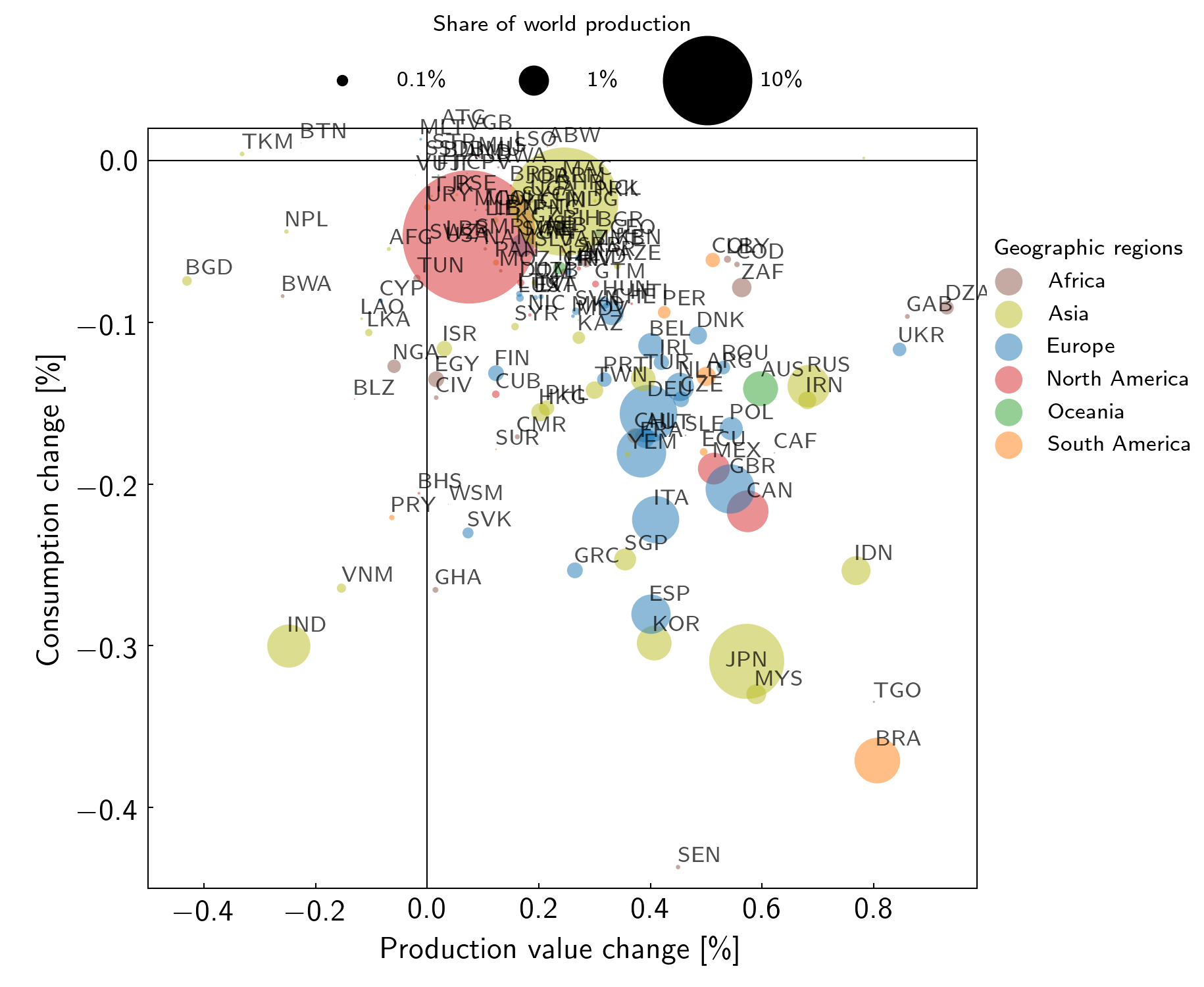

Supplement: S3 Fig — The area of each dot is proportional to the corresponding country’s baseline (unperturbed) production. The dot colors denote the geographic regions (see S2 Table). Quantities are given relative to the baseline (unperturbed) production and consumption, respectively. (TIF) [file pone.0251210.s003.tif]

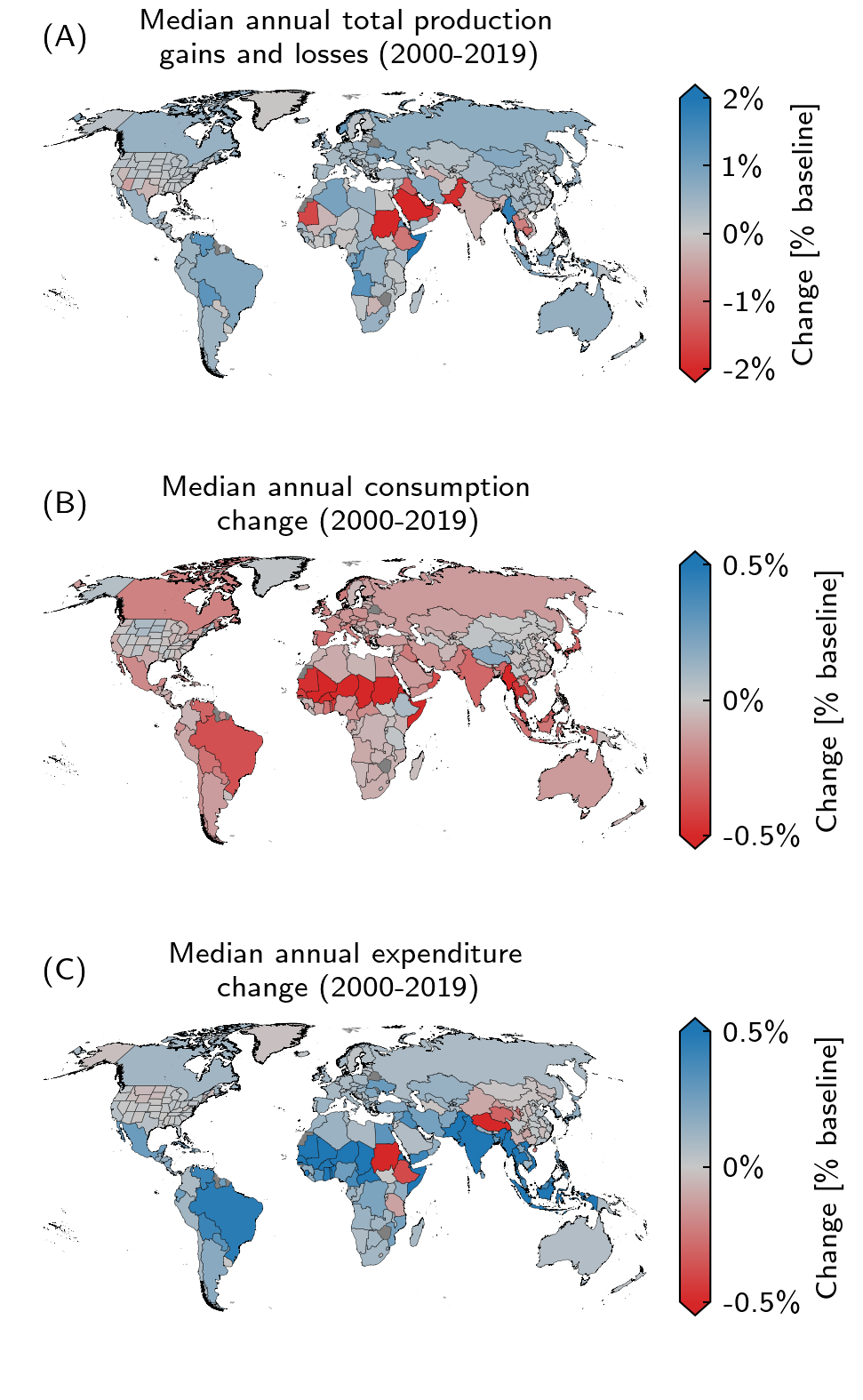

Supplement: S4 Fig — Median annual change of A total production, B consumption and C expenditure relative to the unperturbed baseline. (TIF) [file pone.0251210.s004.tif]
