# Supplementary material for: Future heat stress to reduce people’s purchasing power
Source: PLoS One. 2021 Jun 10;16(6):e0251210. doi: 10.1371/journal.pone.0251210 (PMC8191966; doi:10.1371/journal.pone.0251210)
Supplement: S1 Table — For sectors prone to heat stress-induced productivity loss the respective reduction factor (see Methods) is given in the last column. (PDF) [file pone.0251210.s005.pdf]

S1 Table. Sectors used in the simulations. For sectors prone to heat stress-induced productivity loss the respective reduction factor (see Methods) is given in the last column.

Code	Name	Category	Production reduction factor
AGRI	Agriculture	vital	-0.8 p.p./℃
FISH	Fishing	vital	-0.8 p.p./℃
MINQ	Mining and quarrying	other	-4.2 p.p./℃
GAST	Hotels and restaurants	other	-6.1 p.p./℃
WHOT	Wholesale trade	relevant	-6.1 p.p./℃
OTHE	Others	other	-2.2 p.p./℃
REPA	Maintenance and Repair	other	/
RETT	Retail Trade	relevant	/
FOOD	Food and Beverages	vital	/
TEXL	Textiles and Wearing Apparel	relevant	/
TRAN	Transport	relevant	/
WOOD	Wood and Paper	relevant	/
OILC	Petroleum, Chemical & Non-Metallic Mineral Products	relevant	/
FINC	Financial Intermediation and Business Activities	other	/
METL	Metal Products	relevant	/
MACH	Electrical and Machinery	relevant	/
TREQ	Transport Equipment	relevant	/
MANU	Other Manufacturing	relevant	/
REXI	Re-export and Re-import	other	/
CONS	Construction	relevant	/
ADMI	Public Administration	other	/
EDHE	Education, Health and Other Services	vital	/
HOUS	Private Households	other	/
COMM	Post and Telecommunications	relevant	/
RECY	Recycling	other	/
ELWA	Electricity, Gas and Water	vital	/
FCON	Final consumption	relevant	/
