# Supplementary material for: Future heat stress to reduce people’s purchasing power
Source: PLoS One. 2021 Jun 10;16(6):e0251210. doi: 10.1371/journal.pone.0251210 (PMC8191966; doi:10.1371/journal.pone.0251210)
Supplement: S2 Table — Income level corresponds to Gross National Income per capita (GNIpc) of 2012. (PDF) [file pone.0251210.s006.pdf]

S2 Table. Regions used in the simulations. Income level corresponds to Gross National Income per capita (GNIpc) of 2012.
Income level 1: GNIpc $< 1,305$ USD
Income level 2: $1,036$ USD $<$ GNIpc $< 4,085$ USD
Income level 3: $4,086$ USD $<$ GNIpc $< 12,615$ USD
Income level 4: $12,616$ USD $<$ GNIpc

ISO code	Name	Income level	Geographic region
AFG	Afghanistan	1	Asia
ALB	Albania	3	Europe
DZA	Algeria	3	Africa
AND	Andorra	4	Europe
AGO	Angola	3	Africa
ATG	Antigua and Barbuda	4	North America
ARG	Argentina	3	South America
ARM	Armenia	2	Asia
ABW	Aruba	4	South America
AUS	Australia	4	Oceania
AUT	Austria	4	Europe
AZE	Azerbaijan	3	Asia
BHS	Bahamas	4	North America
BHR	Bahrain	4	Asia
BGD	Bangladesh	1	Asia
BRB	Barbados	4	North America
BLR	Belarus	3	Europe
BEL	Belgium	4	Europe
BLZ	Belize	3	North America
BEN	Benin	1	Africa
BMU	Bermuda	4	North America
BTN	Bhutan	2	Asia
BOL	Bolivia	2	South America
BIH	Bosnia and Herzegovina	3	Europe
BWA	Botswana	3	Africa
BRA	Brazil	3	South America
VGB	British Virgin Islands	4	South America
BRN	Brunei Darussalam	4	Asia
BGR	Bulgaria	3	Europe
BFA	Burkina Faso	1	Africa
BDI	Burundi	1	Africa
KHM	Cambodia	1	Asia
CMR	Cameroon	2	Africa
CAN	Canada	4	North America
CPV	Cabo Verde	2	Africa
CYM	Cayman Islands	4	South America
CAF	Central African Republic	1	Africa
TCD	Chad	1	Africa

ISO code	Name	Income level	Geographic region
CHL	Chile	4	South America
CN.AH	Anhui	3	China, Asia
CN.BJ	Beijing	3	China, Asia
CN.CQ	Chongqing	3	China, Asia
CN.FJ	Fujian	3	China, Asia
CN.GS	Gansu	3	China, Asia
CN.GD	Guangdong	3	China, Asia
CN.GX	Guangxi	3	China, Asia
CN.GZ	Guizhou	3	China, Asia
CN.HA	Hainan	3	China, Asia
CN.HB	Hebei	3	China, Asia
CN.HL	Heilongjiang	3	China, Asia
CN.HE	Henan	3	China, Asia
CN.HU	Hubei	3	China, Asia
CN.HN	Hunan	3	China, Asia
CN.JS	Jiangsu	3	China, Asia
CN.JX	Jiangxi	3	China, Asia
CN.JL	Jilin	3	China, Asia
CN.LN	Liaoning	3	China, Asia
CN.NM	Nei Mongol	3	China, Asia
CN.NX	Ningxia Hui	3	China, Asia
CN.QH	Qinghai	3	China, Asia
CN.SA	Shaanxi	3	China, Asia
CN.SD	Shandong	3	China, Asia
CN.SH	Shanghai	3	China, Asia
CN.SX	Shanxi	3	China, Asia
CN.SC	Sichuan	3	China, Asia
CN.TJ	Tianjin	3	China, Asia
CN.XJ	Xinjiang Uygur	3	China, Asia
CN.XZ	Xizang	3	China, Asia
CN.YN	Yunnan	3	China, Asia
CN.ZJ	Zhejiang	3	China, Asia
COL	Colombia	3	South America
COG	Republic Congo	2	Africa
CRI	Costa Rica	3	North America
HRV	Croatia	4	Europe
CUB	Cuba	3	North America
CYP	Cyprus	4	Europe
CZE	Czech Republic	4	Europe
CIV	Côte d'Ivoire	2	Africa
PRK	North Korea	1	Asia
COD	Democratic Republic Congo	1	Africa
DNK	Denmark	4	Europe
DJI	Djibouti	2	Africa
DOM	Dominican Republic	3	North America
ECU	Ecuador	3	South America

ISO code	Name	Income level	Geographic region
EGY	Egypt	2	Africa
SLV	El Salvador	2	North America
ERI	Eritrea	1	Africa
EST	Estonia	4	Europe
ETH	Ethiopia	1	Africa
FJI	Fiji	3	Oceania
FIN	Finland	4	Europe
FRA	France	4	Europe
PYF	French Polynesia	4	Oceania
GAB	Gabon	3	Africa
GMB	Gambia	1	Africa
GEO	Georgia	2	Asia
DEU	Germany	4	Europe
GHA	Ghana	2	Africa
GRC	Greece	4	Europe
GRL	Greenland	4	North America
GTM	Guatemala	2	North America
GIN	Guinea	1	Africa
GUY	Guyana	2	South America
HTI	Haiti	1	North America
HND	Honduras	2	North America
HKG	Hong Kong	4	Asia
HUN	Hungary	3	Europe
ISL	Iceland	4	Europe
IND	India	2	Asia
IDN	Indonesia	2	Asia
IRN	Iran	3	Asia
IRQ	Iraq	3	Asia
IRL	Ireland	4	Europe
ISR	Israel	4	Asia
ITA	Italy	4	Europe
JAM	Jamaica	3	North America
JPN	Japan	4	Asia
JOR	Jordan	3	Asia
KAZ	Kazakhstan	3	Asia
KEN	Kenya	1	Africa
KWT	Kuwait	4	Asia
KGZ	Kyrgyz Republic	1	Asia
LAO	Lao PDR	2	Asia
LVA	Latvia	4	Europe
LBN	Lebanon	3	Asia
LSO	Lesotho	2	Africa
LBR	Liberia	1	Africa
LBY	Libya	3	Africa
LIE	Liechtenstein	4	Europe
LTU	Lithuania	4	Europe

ISO code	Name	Income level	Geographic region
LUX	Luxembourg	4	Europe
MAC	Macao	4	Asia
MDG	Madagascar	1	Africa
MWI	Malawi	1	Africa
MYS	Malaysia	3	Asia
MDV	Maldives	3	Asia
MLI	Mali	1	Africa
MLT	Malta	4	Europe
MRT	Mauritania	2	Africa
MUS	Mauritius	3	Africa
MEX	Mexico	3	North America
MCO	Monaco	4	Europe
MNG	Mongolia	2	Asia
MNE	Montenegro	3	Europe
MAR	Morocco	2	Africa
MOZ	Mozambique	1	Africa
MMR	Myanmar	1	Asia
NAM	Namibia	3	Africa
NPL	Nepal	1	Asia
NLD	Netherlands	4	Europe
ANT	Netherlands Antilles	4	Sout America
NCL	New Caledonia	4	Oceania
NZL	New Zealand	4	Oceania
NIC	Nicaragua	2	North America
NER	Niger	1	Africa
NGA	Nigeria	2	Africa
NOR	Norway	4	Europe
PSE	West Bank and Gaza	2	Asia
OMN	Oman	4	Asia
PAK	Pakistan	2	Asia
PAN	Panama	3	South America
PNG	Papua New Guinea	2	Asia
PRY	Paraguay	2	South America
PER	Peru	3	South America
PHL	Philippines	2	Asia
POL	Poland	4	Europe
PRT	Portugal	4	Europe
QAT	Qatar	4	Asia
KOR	South Korea	4	Asia
MDA	Moldova	2	Europe
ROU	Romania	3	Europe
RUS	Russian Federation	4	Asia
RWA	Rwanda	1	Africa
WSM	Samoa	2	Oceania
SMR	San Marino	4	Europe
STP	São Tomé and Principe	2	Africa

ISO code	Name	Income level	Geographic region
SAU	Saudi Arabia	4	Asia
SEN	Senegal	2	Africa
SRB	Serbia	3	Europe
SYC	Seychelles	3	Africa
SLE	Sierra Leone	1	Africa
SGP	Singapore	4	Asia
SVK	Slovak Republic	4	Europe
SVN	Slovenia	4	Europe
SOM	Somalia	1	Africa
ZAF	South Africa	3	Africa
SSD	South Sudan	1	Africa
ESP	Spain	4	Europe
LKA	Sri Lanka	2	Asia
SDN	Sudan	2	Africa
SUR	Suriname	3	South America
SWZ	Swaziland	2	Africa
SWE	Sweden	4	Europe
CHE	Switzerland	4	Europe
SYR	Syrian Arab Republic	2	Asia
TWN	Taiwan	4	Asia
TJK	Tajikistan	1	Asia
THA	Thailand	3	Asia
MKD	Macedonia	3	Europe
TGO	Togo	1	Africa
TTO	Trinidad and Tobago	4	South America
TUN	Tunisia	3	Africa
TUR	Turkey	3	Asia
TKM	Turkmenistan	3	Asia
UGA	Uganda	1	Africa
UKR	Ukraine	2	Europe
ARE	United Arab Emirates	4	Asia
GBR	United Kingdom	4	Europe
TZA	Tanzania	1	Africa
US.AL	Alabama	4	USA, North America
US.AK	Alaska	4	USA, North America
US.AZ	Arizona	4	USA, North America
US.AR	Arkansas	4	USA, North America
US.CA	California	4	USA, North America
US.CO	Colorado	4	USA, North America
US.CT	Connecticut	4	USA, North America
US.DE	Delaware	4	USA, North America
US.DC	District of Columbia	4	USA, North America
US.FL	Florida	4	USA, North America
US.GA	Georgia	4	USA, North America
US.HI	Hawaii	4	USA, North America
US.ID	Idaho	4	USA, North America

ISO code	Name	Income level	Geographic region
US.IL	Illinois	4	USA, North America
US.IN	Indiana	4	USA, North America
US.IA	Iowa	4	USA, North America
US.KS	Kansas	4	USA, North America
US.KY	Kentucky	4	USA, North America
US.LA	Louisiana	4	USA, North America
US.ME	Maine	4	USA, North America
US.MD	Maryland	4	USA, North America
US.MA	Massachusetts	4	USA, North America
US.MI	Michigan	4	USA, North America
US.MN	Minnesota	4	USA, North America
US.MS	Mississippi	4	USA, North America
US.MO	Missouri	4	USA, North America
US.MT	Montana	4	USA, North America
US.NE	Nebraska	4	USA, North America
US.NV	Nevada	4	USA, North America
US.NH	New Hampshire	4	USA, North America
US.NJ	New Jersey	4	USA, North America
US.NM	New Mexico	4	USA, North America
US.NY	New York	4	USA, North America
US.NC	North Carolina	4	USA, North America
US.ND	North Dakota	4	USA, North America
US.OH	Ohio	4	USA, North America
US.OK	Oklahoma	4	USA, North America
US.OR	Oregon	4	USA, North America
US.PA	Pennsylvania	4	USA, North America
US.RI	Rhode Island	4	USA, North America
US.SC	South Carolina	4	USA, North America
US.SD	South Dakota	4	USA, North America
US.TN	Tennessee	4	USA, North America
US.TX	Texas	4	USA, North America
US.UT	Utah	4	USA, North America
US.VT	Vermont	4	USA, North America
US.VA	Virginia	4	USA, North America
US.WA	Washington	4	USA, North America
US.WV	West Virginia	4	USA, North America
US.WI	Wisconsin	4	USA, North America
US.WY	Wyoming	4	USA, North America
URY	Uruguay	4	South America
UZB	Uzbekistan	2	Asia
VUT	Vanuatu	2	Oceania
VEN	Venezuela	3	South America
VNM	Vietnam	2	Asia
YEM	Yemen	2	Asia
ZMB	Zambia	2	Africa
ZWE	Zimbabwe	1	Africa
