# Supplementary material for: Future heat stress to reduce people’s purchasing power
Source: PLoS One. 2021 Jun 10;16(6):e0251210. doi: 10.1371/journal.pone.0251210 (PMC8191966; doi:10.1371/journal.pone.0251210)
Supplement: S3 Table — Values are based on GTAP [55]. (PDF) [file pone.0251210.s007.pdf]

S3 Table. Consumption price elasticities per income level and sector category. Values are based on GTAP.

	Income level			
Sector category	1	2	3	4
vital	-0.15	-0.2	-0.3	-0.45
relevant	-0.2	-0.3	-0.4	-0.65
other	-0.3	-0.4	-0.5	-0.75
